# Fatty Acid Binding Protein 6 Inhibition Decreases Cell Cycle Progression, Migration and Autophagy in Bladder Cancers

**DOI:** 10.3390/ijms23042154

**Published:** 2022-02-15

**Authors:** Chieh-Hsin Lin, Hsin-Han Chang, Chien-Rui Lai, Hisao-Hsien Wang, Wen-Chiuan Tsai, Yu-Ling Tsai, Chih-Ying Changchien, Yu-Chen Cheng, Sheng-Tang Wu, Ying Chen

**Affiliations:** 1 National Defense Medical Center, Department of Biology and Anatomy, Taipei 11490, Taiwan; jessie19950403@gmail.com (C.-H.L.); albertchang1008@gmail.com (H.-H.C.); ray42904917@gmail.com (C.-R.L.); koala8072@yahoo.com.tw (C.-Y.C.); plokmijzz@gmail.com (Y.-C.C.); 2 Department of Urology, Cheng Hsin General Hospital, Taipei 11490, Taiwan; hsiao386@ms13.hinet.net; 3 National Defense Medical Center, Department of Pathology, Tri-Service General Hospital, Taipei 11490, Taiwan; ab95057@hotmail.com (W.-C.T.); c909228@gmail.com (Y.-L.T.); 4 National Defense Medical Center, Department of Internal Medicine, Tri-Service General Hospital, Taipei 11490, Taiwan; 5 National Defense Medical Center, Division of Urology, Department of Surgery, Tri-Service General Hospital, Taipei 11490, Taiwan

**Keywords:** FABP6, bladder cancer, proliferation, cell cycle, migration, autophagy

## Abstract

Bladder cancer (BC) has a high recurrence rate worldwide. The aim of this study was to evaluate the role of fatty acid binding protein 6 (FABP6) in proliferation and migration in human bladder cancer cells. Cell growth was confirmed by MTT and colony formation assay. Western blotting was used to explore protein expressions. Wound healing and Transwell assays were performed to evaluate the migration ability. A xenograft animal model with subcutaneous implantation of BC cells was generated to confirm the tumor progression. Knockdown of FABP6 reduced cell growth in low-grade TSGH-8301 and high-grade T24 cells. Cell cycle blockade was observed with the decrease of CDK2, CDK4, and Ki67 levels in FABP6-knockdown BC cells. Interestingly, knockdown of FBAP6 led to downregulation of autophagic markers and activation of AKT-mTOR signaling. The application of PI3K/AKT inhibitor decreased cell viability mediated by FABP6-knockdown additionally. Moreover, FABP6-knockdown reduced peroxisome proliferator-activated receptor γ and retinoid X receptor α levels but increased p-p65 expression. Knockdown of FABP6 also inhibited BC cell motility with focal adhesive complex reduction. Finally, shFABP6 combined with cisplatin suppressed tumor growth in vivo. These results provide evidence that FABP6 may be a potential target in BC cells progression.

## 1. Introduction

Bladder cancer (BC) is the 10th most-commonly diagnosed cancer worldwide, with high mortality and recurrence rates [1]. BC can be divided into non-muscle invasive BC (NMIBC, Ta and T1) and muscle invasive BC (MIBC, T2 and T3). Approximately 75% of BC patients are considered as NMIBC—the most prevalent type of BC. Transurethral resection of bladder tumor (TURBT) followed by immediate postoperative instillation of intravesical chemotherapy then adjuvant intravesical immunotherapy (BCG) or chemotherapy is the standard of care for NMIBC. Chemotherapy with gemcitabine and cisplatin is commonly used in muscle-invasive BC [2]. Despite state-of-the-art surgical techniques and neoadjuvant chemotherapy, the five-year survival rate is approximately 60% in MIBC [3,4]. Consequently, there is an urgent need to find new targets for BC therapy to improve the outcome of survival and recurrence rates in patients with BC.

Fatty acid binding proteins are intracellular lipid-binding proteins that act as chaperones for lipids. These proteins bind lipids in different organelles and regulate metabolic homeostasis through lipid signal transduction [5]. They are abundantly expressed in specific tissues, such as the liver and small intestine, where fatty acids are the dominant material for lipid metabolism. Fatty acid binding protein 6 (FABP6) is also known as ileal bile acid-binding protein (IBABP). It is known for its unique ligand, bile acid, and directly governs the ileal bile acid transport system [6]. For all FABPs, there is a wealth of literature focusing on interrelations involving transcription factors, such as peroxisome proliferator-activated receptors (PPARs). PPARs encompass a DNA-binding domain that controls gene expression. PPARs play a vital role in the regulation of lipid metabolism and may be related to FABPs. Previous research surrounding FABP6 has indicated its overexpression in colorectal cancers [7] and associated with the prognosis of pancreatic cancer [8]. However, the role of FABP6 has not been explored in urothelial carcinoma (UC).

Dysregulation of the cell cycle usually causes abnormal proliferation of cancer cells, and the link to genetic instability usually plays a pivotal role in the transformation of normal cells into cancerous ones. In BC, dysregulated cell cycle has been proven to contribute to carcinogenesis of the urothelial epithelium [9]. The current study aimed to investigate the role of FABP6 in human bladder cancer cells. Therefore, TSGH-8301 and T24 were applied as low- and high-grade BC cell lines, respectively. The role of urothelial FABP6 was examined with respect to cell proliferation and migration. This may represent promise for BC patient therapy and highlight potential targets to improve the development of BC treatment modalities.

## 2. Results

### 2.1. Expression Pattern of FABP6 in Uroepithelium, Low- and High-Grade Urothelial Carcinomas

**Figure 1:**
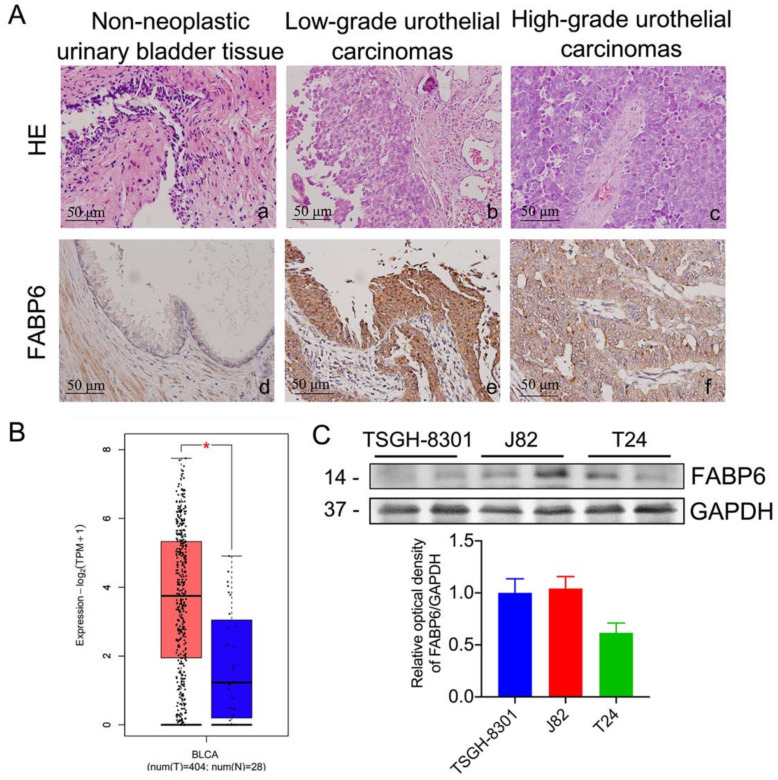
FABP6 expression in BC cells. (**A**) Hematoxylin and eosin (H&E) staining (a to c, upper column) of normal urinary bladder tissue, low-grade urothelial carcinoma, and high-grade urothelial carcinoma. Immunohistological analysis of FABP6 in the lower column (d to f, original magnification was ×400). (**B**) The correlation of FABP6 expression with survival time was analyzed using the Gene Expression Profiling Interactive Analysis (GEPIA) website. The blue column shows normal bladder tissue (n = 28), and the red column displays BC tissue (n = 404). *, *p* < 0.05 compared to normal tissue. (**C**) FABP6 protein expression was detected by western blotting in low-grade TSGH-8301, and high-grade J82 and T24 cells. GAPDH was a loading control.

H&E staining revealed representative images of non-neoplastic uroepithelium, low- and high-grade urothelial carcinomas (UC) (Figure 1A). Corresponding immunohistochemistry sections had enhanced FABP6 staining in both low- and high- grade UC. In addition, bladder neoplasms (orange) showed higher FABP6 expression levels than normal tissues (blue) in the Gene Expression Profiling Interactive Analysis (GEPIA) [10] (Figure 1B). FABP6 expression in different BC cell lines was analyzed by Western blotting (Figure 1C). The above results indicated increased FABP6 expression in bladder neoplasms.

### 2.2. Knockdown of FABP6 Inhibited Survival and Colony Formation in BC Cells

**Figure 2:**
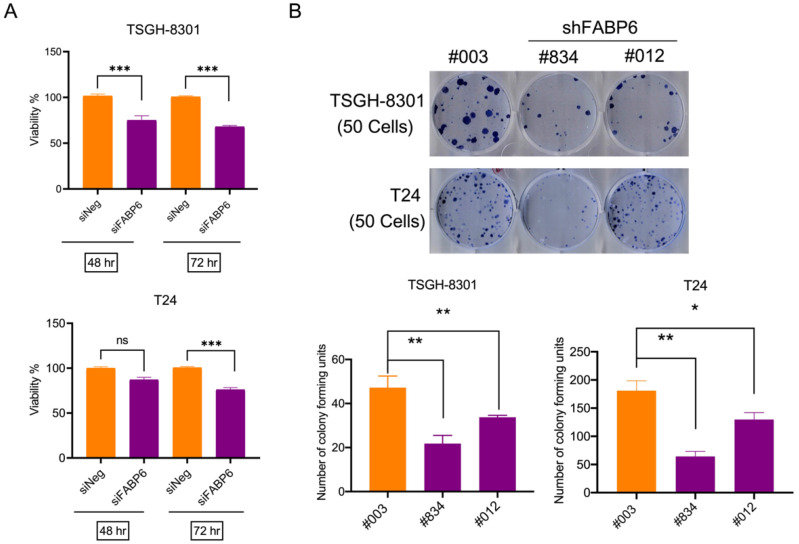
Downregulating FABP6 expression decreased cell viability. (**A**) The survival rates of TSGH-8301 and T24 cells were analyzed after siFABP6 transfection for 48 and 72 h using MTT assays. ns showed not significant compared to control group. (**B**) Colony formation assays for TSGH-8301 and T24 cells were analyzed after lentiviral infection of shFABP6 (#834 and #012). Cell colonies were further visualized after Coomassie blue staining and quantified after 10 days of incubation. *, *p* < 0.05; **, *p* < 0.01; ***, *p* < 0.001 compared to control group.

The MTT assay was used to evaluate the effects of siFABP6 on the survival of human BC cells. The knockdown effect of siFABP6 in BC was shown in Supplementary Figure S1A,B. After 72 h transfected with siFABP6, reductions in viability of TSGH-8301 and T24 cells were observed by 33% and 25%, respectively (Figure 2A). Subsequently, colony formation of TSGH-8301 and T24 cells infected with lentiviral-shFABP6 or Scramble Control was evaluated. The knockdown effect of shFABP6 in BC was shown in Supplementary Figure S1C. The results showed that the colony number was reduced following FABP6 knockdown (Figure 2B). These results indicated that downregulation of FBAP6 in UC cells inhibited cell survival and colony formation abilities.

### 2.3. Knockdown of FABP6 induced Cell Cycle Arrest of BC Cells

**Figure 3:**
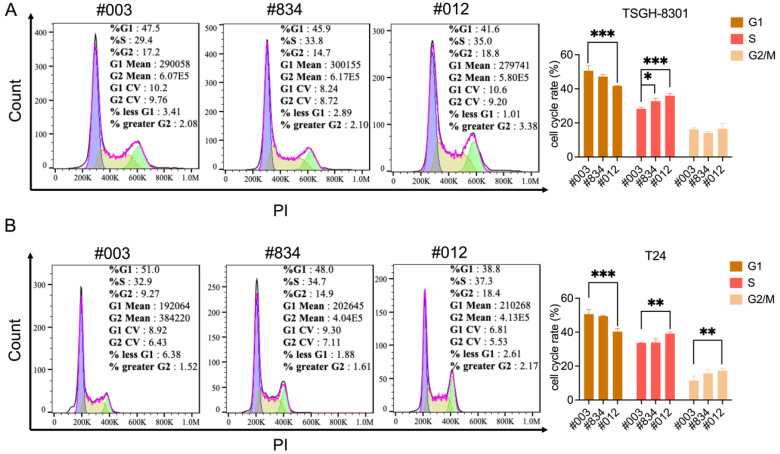
Knockdown of FABP6 induced S and G2/M phase cell-cycle arrest in BC cells. Cell-cycle status was investigated using PI staining after knockdown of FABP6 in (**A**) TSGH-8301 and (**B**) T24 cells. Cells were treated with 1 mg/mL doxycycline for three days, and then cells were collected and fixed in 70% alcohol. Then, cells were stained with PI and analyzed by flow cytometry. 10,000 cells were acquired for analysis. *, *p* < 0.05; **, *p* < 0.01; ***, *p* < 0.001 compared to control group.

Cell cycle regulation is critical for cancer cell viability. Cell cycle distribution was detected by flow cytometry after FABP6 reduction. The results showed that knockdown of FABP6 led to accumulation in the S and G2/M phases in TSGH-8301 and T24 cells (Figure 3A,B). As shown in Figure 3A, quantitative analysis of S phase distribution of TSGH-8301 cells increased to 32.7% and 35.9% in the shFABP6 group compared to the shControl group (28.2%). In contrast, both S and G2/M arrest was observed in T24 cells. As shown in Figure 3B, the G2/M phase and S phase distribution significantly increased to 17.2% and 39.1%. We further investigated the mechanism of cell cycle arrest in BC cells.

**Figure 4:**
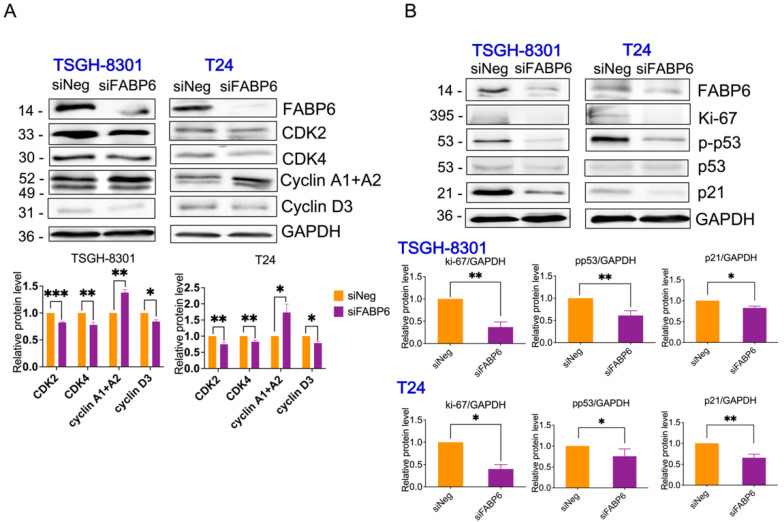
Effects of FABP6 inhibition on cell-cycle related protein expression. (**A**) Western blot analysis of CDK2, CDK4, cyclin A, and cyclin D3 expression after knockdown of FABP6 for 72 h in TSGH-8301 and T24 cells. (**B**) Western blot analysis of p-p53, p53, p21 and ki-67 expression after knockdown of FABP6 for 72 h in TSGH-8301 and T24 cells. GAPDH was a loading control. *, *p* < 0.05; **, *p* < 0.01; ***, *p* < 0.001 compared to siNegative control group (siNeg).

In both TSGH8301 and T24 cells, the protein levels of CDK2, CDK4, and cyclin D3 were significantly downregulated (Figure 4A). Moreover, the expression of p-p53, p21, and Ki-67 proteins was decreased following FABP6 knockdown (Figure 4B and Supplementary Figure S3). Thus, the knockdown of FABP6 inhibited proliferation via cell-cycle arrest in BC. Apoptosis was not induced by FABP6 inhibition in BC cells with no cleaved caspase 3 and PARP being observed (Supplementary Figure S2).

### 2.4. Reducing of FABP6 Inhibited Autophagy Markers via Akt/mTOR Pathway

**Figure 5:**
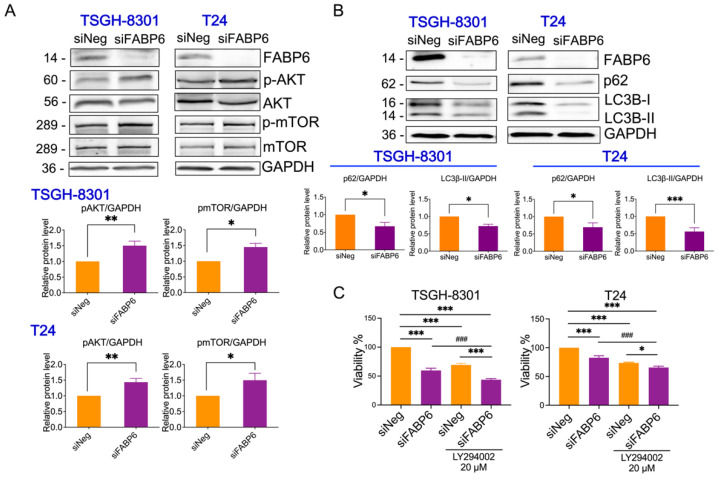
Effects of *FABP6* knockdown on mTOR/Akt and autophagy in BC cells. (**A**) Western blotting analysis of p-AKT, AKT, p-mTOR, and mTOR after knockdown of *FABP6* for 72 h in TSGH-8301 and T24 cells. (**B**) Western blotting analysis of expression of p62 and LC3B-I/LC3B-II after knockdown of *FABP6* for 72 h. Left panel: TSGH-8301 cell line; right panel: T24 cell line. (**C**) PI3K inhibitor LY294002 (20 μM) or DMSO were co-treated with siFABP6 for 72 hr Cell survival was measured using MTT assays. *, *p* < 0.05; **, *p* < 0.01; ***, *p* < 0.001 compared to siNeg group. ###, *p* < 0.001 compared to siFABP6 group.

FABPs have also been found to be correlated with autophagy under ER stress and mitochondrial stress [11]. After knockdown of FABP6 for 72 h, p-AKT and p-mTOR were upregulated in both TSGH8301 and T24 cell lines (Figure 5A,B). As shown in Figure 5C,D, p62 protein, which binds to LC3B leading to autophagosome degradation [12] was downregulated by FABP6 reduction, which may lead to blockage of autophagy. In addition, the mRNA level of p62 was unaffected while FABP6 was inhibition (Supplementary Figure S4B). The levels of the autophagy marker, LC3B-II, were lower in the siFABP6 group than in the control group (Figure 5C,D). Intriguingly, the PI3K inhibitor LY294002 showed an additional inhibitory effect of cell growth by FABP6 knockdown (Figure 5E,F). Moreover, application of LY294002 could not reverse the reduction of p62 and LC3B-II (Supplementary Figure S4). Together, these results may suggest that knockdown of FABP6 suppressed autophagic markers.

Additionally, the proteasome inhibitor MG132 and the lysosomal inhibitor NH4Cl were employed to investigate whether degradation systems involved in FABP6 reduction inhibited LC3B-II expression. Only MG132 recovered the expression of LC3B-II in FABP6 knockdown cells (Supplementary Figure S3A). MTT assays further showed that regardless of the cell line, treatment with MG132 led to an additional effect of reduced cell survival (Supplementary Figure S5B).

### 2.5. FABP6 Inhibition Decreased Nuclear Receptor PPARγ/RXRα and Activated NF-kB Signaling

**Figure 6:**
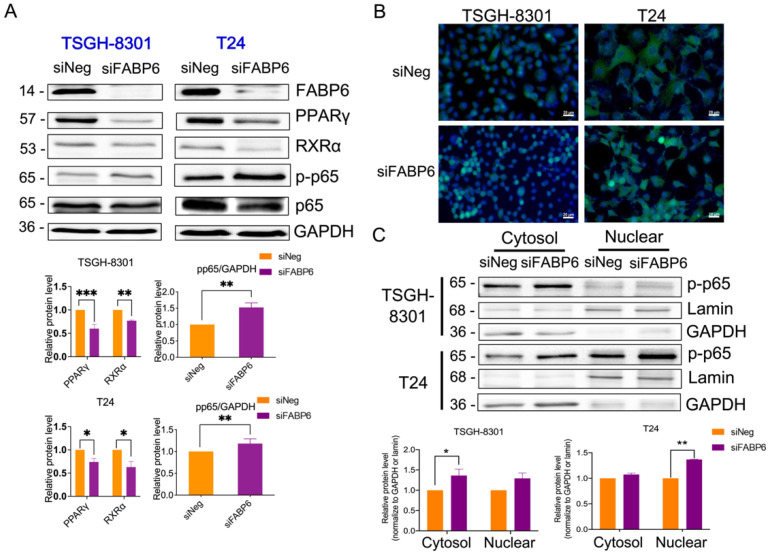
Effects of FABP6 knockdown on PPARγ and NF-κB signaling pathways. (**A**) Expression of PPARγ, RXRα, p-p65, and p65 was analyzed after FABP6 knockdown for 72 h. GAPDH was a loading control. (**B**) Immunostaining of p-p65 after siFABP6 72 h post-transfection. DAPI was counter stained for nucleus. Scale bar = 20 μm. (**C**) Nuclear and cytosolic p-p65 expression was analyzed by nuclear fractionation. Lamin and GAPDH served as nuclear and cytosolic loading controls, respectively. *, *p* < 0.05; **, *p* < 0.01; ***, *p* < 0.001 compared to siNeg group.

PPARs are nuclear receptors that combine with retinoid X receptors (RXRs) to be sensors of lipid signaling in the context of autophagy regulation [13]. The protein levels of PPARγ and RXRα were reduced after the knockdown of FABP6 in both cell lines (Figure 6A). Immunostaining of p-p65 observed an upregulation after FABP6 inhibition (Figure 6B). Thereafter, we examined the total, nuclear, and cytosolic protein levels of NF-κB. As expected, the results showed an increase in NF-κB levels after FABP6 silencing (Figure 6C). Accordingly, FABP6 inhibition induced NF-κB activation, which might be antagonized by PPARγ modulation.

### 2.6. Reducing of FABP6 Decreased Cell Migration and Invasion Characteristics in siFABP6-Transfected TSGH-8301 and T24 Cells

**Figure 7:**
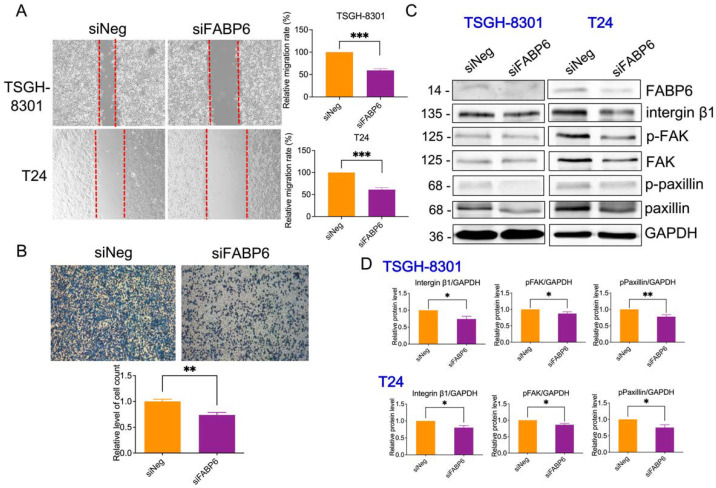
Effect of *FABP6* knockdown on cell migration and invasion in BC cells. (**A**) Cell migration was detected using wound healing assays in siNeg and *siFABP6* groups 72 h post-transfection. 8 h after scratching, the wound area was captured and analyzed using ImageJ software. (**B**) Cell invasion was detected using Transwell migration assays using Matrigel. After *siFABP6* transfection for 72 h, T24 cells were incubated in the upper chamber. After cells were seeded on the Matrigel for 24 h, cells in the lower chamber were observed by Coomassie blue staining and counted using ImageJ software. (**C**,**D**) The expression of integrin β1, p-FAK, FAK, p-paxillin, and paxillin was analyzed after *FABP6* knockdown for 72 h. GAPDH was a loading control. *, *p* < 0.05; **, *p* < 0.01; ***, *p* < 0.001 compared to siNeg group.

To determine whether FABP6 is involved in BC cell migration and invasion, wound healing assays and transwell assays were performed. Compared with the control group, cell migration was decreased by 40% ± 4% in both TSGH-8301 and T24 cells after wounding (Figure 7A). The number of invasive T24 cells declined considerably to 73% compared to the siNegative control group (Figure 7B). In addition, focal adhesive proteins, including integrin β1, p-focal adhesion kinase (p-FAK), and p-paxillin were reduced by FABP6-knockdown in both cell lines (Figure 7C). In addition, E-cadherin and N-cadherin were unaffected after FABP6 knockdown, except the reduction of E-cadherin in T24 cells (Supplementary Figure S6). Our findings suggested that FABP6-knockdown decreased the migration and invasive capabilities of TSGH-8301 and T24 cells.

### 2.7. FABP6-Knockdown Suppressed BC Cell Growth In Vivo

**Figure 8:**
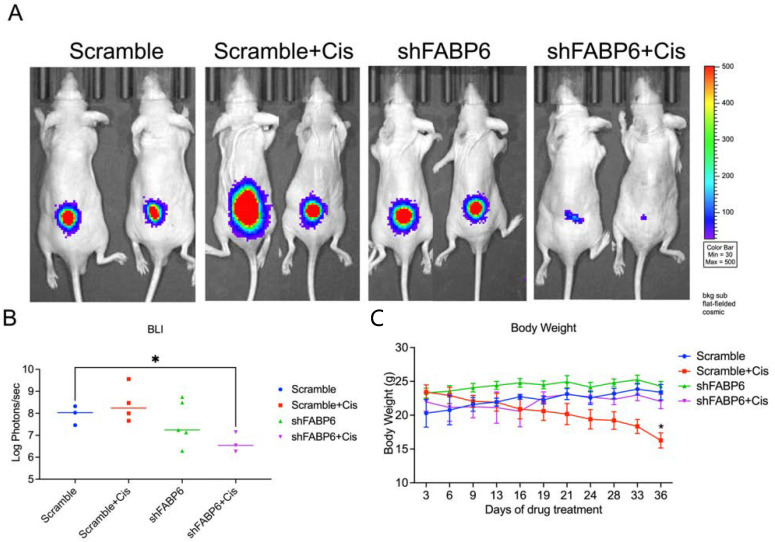
Knockdown of *FABP6* suppressed BC cell growth in vivo. BALB/c nude mice were subcutaneously injected with T24 cells and allowed to grow for 3 weeks. Cisplatin (2.5 mg/kg) and vehicle were then intraperitoneally injected every 3 days for 3 weeks. (**A**) Tumor volumes were measured using IVIS spectrum every 3 days before and during drug administration. The IVIS images were shown each group at five weeks. (**B**) Luminescent intensity of photons emitted from each tumor was quantified. (**C**) Body weight was measured every 3 days. *, *p* < 0.05 compared with scramble control group.

We established a mouse model by subcutaneous injection of T24 cells to further evaluate the effect of FABP6 on BC cell growth in vivo (Figure 8A). The results suggested that knockdown of FABP6 combined with cisplatin (2.5 mg/kg) treatment remarkably suppressed tumor growth after five weeks as shown by BLI value (Figure 8B). Loss of body weight was observed in the cisplatin treatment groups compared to that in the non-cisplatin-based groups (Figure 8C). These results indicated that combination of FABP6 inhibition and cisplatin suppressed BC cell progression.

## 3. Discussion

FABP6 inhibition blocked cell proliferation via cell-cycle arrest. CDK2 and CDK4 reduction arrest the G1 cell-cycle in MCF-7 human breast cancer cells [14]. Cyclin D3 is essential for the G1/S transition [15]. Additionally, cyclin D3 has been reported to be used as a predictive marker in breast cancer, and its reduction decreases malignant cell migration and invasion [16]. However, the driving force to pass the G2 checkpoint in human cells depends on p53 and p21 [17]. P21 inhibits CDK2 and CDK4 by blocking pRb phosphorylation [18]. According to our results, suppression of FABP6 expression in BC decreased the expression of p53, p21, CDK2, and CDK4. Abrogation of FABP6 expression increased cell-cycle arrest in the G2/M phase, which may be due to CDK2, CDK4, and cyclin D3 reductions in BC.

Mice treated with cisplatin (4 mg/Kg) show significantly reduced body weights from week 2 to week 6 compared to a saline group [19]. Previous studies also showed that administrating cisplatin to mice significantly reduced their body weight [20,21,22]. A lower body weight in the cisplatin treatment group than in control group is also observed in breast cancer-bearing mice [23]. Accordingly in our study, the body weight was lower in the cisplatin treatment group than in the control group.

P62 plays a critical role in autophagy, proliferation, and migration. Moreover, p62 has been reported to modulate endogenous and environmental stress [24]. When the autophagic flux is inhibited by chloroquine or bafilomycin A1, both p62 and LC3B-II accumulation are observed [25]. In contrast, the p62 protein expression was decreased after FABP6 reduction in BC cells without mRNA levels changing. Meanwhile, LC3B-II was downregulated by FABP6 knockdown. On the other hand, knockdown of p62 inhibits proliferation and migration in lung adenocarcinoma [26]. Accordingly, the autophagic flux, proliferation, and migration might be inhibited by p62 reduction in FABP6 knockdown BC cells.

Activation of the Akt/mTOR pathway serves as a negative regulator of autophagy [27]. Pharmacological inhibition of autophagy was found to induce cytotoxicity with accumulation of cellular stress markers in leukemia, breast cancer, and colon cancer cells [28]. In addition, p62 protein, a component of the autophagosome, is frequently upregulated following deficient autophagy [29]. The current study of *FABP6*-knockdown in BC revealed increased p-Akt and p-mTOR protein levels with decreased autophagy marker p62 and LC3B-II. Previous studies have characterized the potential of autophagy inhibitors in the treatment of BC by enhancing chemotherapy sensitivity [30]. After knocking-down *FABP6*, an increase in autophagy was not observed, but, rather, showed deficiency, suggesting that autophagy may protect BC cells from cell cycle-arrest or even cell death. Defective autophagy may cause metabolic disorders, and thus autophagy probably plays a pro-survival role in this scenario. Targeting FABP6 may serve as a therapeutic opportunity for regulating BC cell viability, cell-cycle, and cell autophagy.

Different FABP isoforms have distinct functions in PPARs [31]. PPARγ expression is correlated with inflammation, lipid, and cellular metabolism [32,33]. Additionally, PPAR and RXR heterodimers have been associated with *FABP1* and *FABP4* transcription [33]. Activation of FABP1 in HepG2 cells induces *PPARα* and *PPARγ* transactivation [34]. PPARγ is strongly enhanced in *FABP4*-null preadipocytes, which leads to adipogenesis [35]. In contrast, Src inhibition decreases PPARγ expression and subsequently reduces FABP4, which blocks cell survival in lung cancer cells [36]. Therefore, FABPs may contribute to the expression of PPAR. Moreover, PPARγ activation leads to the repression of pro-inflammatory genes and inhibits the expression of cytokines by inhibiting the NF-κB pathway [37]. According to our results, suppression of FABP6 expression in BC decreased PPARγ levels and RXRα, which may lead to NF-κB activation. 

## 4. Materials and Methods

### 4.1. Cell Culture 

The bladder cancer cell line Tri-Service General Hospital-8301 (TSGH-8301) was provided by Dah-Shyong Yu, M.D. from the Division of Urology, Tri-Service General Hospital, National Defense Medical Center (Taipei, Taiwan). The TSGH-8301 cell line was grown in RPMI 1640 medium. T24 was grown in McCoy’s 5a medium and obtained from the Bioresource Collection and Research Center (BCRC, Hsinchu, Taiwan). J82 cells were obtained from ATCC and grown in RPMI 1640 medium. These cell lines were supplemented with 10% fetal bovine serum (FBS) (Thermo Fisher Scientific, Waltham, MA, USA), L-Glutamine, and sodium pyruvate (Corning, Corning, NY, USA) in a sealed incubator at 37 °C and 5% CO_2_.

### 4.2. Small Interfering RNA (siRNA) Transfection

TSGH-8301 and T24 cells were plated at a density of 5 × 10^5^ cells per well in 6-well plates. The next day, the cells were transfected with FABP6 siRNA (50 nM; siGENOME SMARTPool) or non-targeting control siRNA (Horizon Discovery, Cambridge, UK) using Lipofectamine 3000 reagent (Thermo Fisher Scientific) according to the manufacturer’s instructions for 48 or 72 h of transfection. The siRNA sequences were showed in Supplementary Table S1.

### 4.3. Short Hairpin RNA (shRNA) Transfection

Due to applying the knockdown FABP6 in animal model, we developed the inducible shFABP6 stable knockdown cell line which could be induced by doxycycline. The lentivirus expression plasmids pLKO-shScramble (ASN0000000003) and pLKO-shFABP6 (TRCN0000419834, and TRCN0000447012) were purchased from the National RNAi Core Facility at Academia Sinica (Taipei, Taiwan). The sequences were showed in Supplementary Table S2.

### 4.4. Lentivirus Production

The lentivirus production was by 293T cells transfected pLKO-shScramble or pLKO-shFABP6 with pCMV-dR8.91, and pMD.G using Lipofectamine 3000 reagent (Thermo Fisher Scientific) according to the manufacturer’s instructions. The lentivirus was collected after 24- and 52-h transfection and was used to infect TSGH-8301 and T24 cells, followed by puromycin (2 µg/mL) selection.

### 4.5. MTT Assays

Cells were seeded in 96-well culture plates at a density of 5 × 10^3^ cells/well. To analyze cell survival under knockdown of *FABP6* in TSGH-8301 and T24 cells, different siRNA or vehicle-only control groups were transfected for 48 or 72 h. Next, MTT reagent (3-(4,5-dimethyl-2-thiazolyl)-2,5-diphenyl-2H-tetrazolium bromide, Sigma-Aldrich, Brulington, MA, USA) was added to each well and incubated for 3 h. The MTT solution was removed, followed by the addition of dimethyl sulfoxide (DMSO, Sigma) to dissolve insoluble formazan. The optical absorbance was measured at 570 nm using an ELISA reader Synergy HTX (BioTek Instruments, Winooski, VT, USA).

### 4.6. Flow Cytometric Cell-Cycle Analysis

TSGH-8301 and T24 cells were seeded in 6 cm dishes at 4 × 10^5^ cells/well and 3 × 10^5^ cells/well, respectively. After induction for 3 days, the cells were harvested and washed with PBS. The cells were collected and fixed in 70% alcohol. After fixation, RNase was added and the cells were stained with propidium iodide (PI) dye (Sigma). Cell-cycle distribution was analyzed using Attune NxT Flow Cytometer (Thermo Fisher Scientific) and FlowJo software (BD Biosciences, Franklin Lakes, NJ, USA).

### 4.7. Western Blotting

4 × 10^5^ cells/well of TSGH-8301 cells or 3 × 10^5^ cells/well of T24 cells were seeded in 6 cm plates. Cells were treated with mammalian protein extraction buffer (Cytiva, Marlborough, MA, USA) with phosphatase and protease inhibitor cocktail EDTA free (MedChemExpress, Monmouth Junction, NJ, USA) to dissociate cells from the plate using cell scrapers. A Q700 Sonicator (Qsonica, Newtown, CT, USA) was used to disrupt cell membranes and to obtain a protein extract solution by centrifugation. Protein concentrations were measured using a protein assay kit (Bio-Rad, city, Hercules, CA, USA). 5× SDS Loading Sample buffer (30% glycerol, 10% sodium dodecyl sulfate, 5% β-mercaptoethanol, 250 mM Tris-HCL, pH 6.8, and 0.02% bromophenol blue) was added in different amounts based on the different protein concentrations. Next, 60 or 80 µg of protein samples was electrophoresed on 10% acrylamide gels by SDS-PAGE. Protein samples were then transferred to nitrocellulose membranes (Bio-Rad). The membranes were blocked in blocking buffer and incubated with primary antibodies at 4 °C overnight. The membranes were washed with TBST buffer (Tris-base, sodium chloride, Tween 20) and incubated with HRP-conjugated secondary antibody (Cell Signaling Technology, Danvers, MA, USA) (antibody dilution 1:5000 in blocking buffer) at room temperature. Chemiluminescent detection reagents (Bio-Rad) were applied to the blots, and protein signals were detected by Xplorer (SPOT Imaging, Sterling Heights, MI, USA). The antibodies used are listed in Supplementary Table S3.

### 4.8. Wound Healing Scratch Assay

To analyze cell migration under FABP6 knockdown in TSGH-8301 and T24 cells, different siRNA or vehicle-only groups were transfected for 72 h. After transfection, a wound was scratched into confluent cell monolayers using a 200 µL pipette tip. Cells were washed with PBS 3 times, aspirated, and RPMI-1640 medium was replaced. Wound width images were captured using a microscope at 10× objective magnification (100× total magnification) 0 and 8 h after scratching, and the wound areas were analyzed using ImageJ software. 

### 4.9. Transwell Assays

T24 cells were seeded in the upper chamber of a Transwell at a density of 5 × 10^4^ cells/well Corning. Before seeding, 3% Matrigel mixed with McCoy’s 5A medium was added to the upper chamber and incubated for at least 2 h at 37 °C. After incubation at 37 °C for 24 h, cells in the lower chamber were fixed with formalin and stained with Coomassie Brilliant Blue G250 (Sigma). The invading cells were enumerated using three random microscope fields from each transwell in each experiment.

### 4.10. Xenograft Mouse Model

All animal experiments were approved by the Laboratory Animal Center of the National Defense Medical Center, Taiwan (IACUC No. 19-157). BALB/c nude mice (18 weeks, 20–25 g) were purchased from BioLASCO (Taipei, Taiwan). The animals were anesthetized with an O_2_/isoflurane mixture. Next, 1 × 10^6^ T24-Luc2 cells with inducible knockdown of FABP6 or a scrambled control were implanted subcutaneously into the right flank of mice. Three weeks after implantation, the twenty mice were assigned to four groups (n = 5 each group): vehicle-only control, shFABP6, vehicle control + cisplatin, and shFABP6 + cisplatin. Cisplatin was administered via intraperitoneal injection at 2.5 mg/kg every 3 days for 5 weeks. Doxycycline (2 mg/mL) in drinking water with 0.1% sucrose was replenished once every 2 days after implantation. Body weight was measured once every three days, and a non-invasive in vivo imaging system (IVIS Spectrum, Perkin-Elmer, Waltham, Massachusetts, USA) detection for tumor volume was used every 3 days. After 5 weeks experimental period, vehicle-only control group left three animals, vehicle control + cisplatin group left four animals, and shFABP6 + cisplatin group left three animals. The mice were sacrificed then.

### 4.11. Statistical Analysis

Data are presented as means ± SEM  from at least three independent experiments. Differences between means were assessed using the Kruskal–Wallis test. The Mann–Whitney U test was used for post-hoc analysis. Statistical significance was set at *p* < 0.05.

## 5. Conclusions

FABP6 inhibition reduced cell growth by arresting the cell-cycle with decreased CDK2 and CDK4 abundance, and cyclin D3 expression. AKT/mTOR was activated by *FABP6*-knockdown; however, blocking AKT signaling could not rescue FABP6-reduced cell viability. Moreover, declines in PPARγ and RXRα levels were observed after *FABP6* knockdown. Additionally, FABP6 inhibition also reduced migration, with a decrease in focal adhesive complexes. Thus, FABP6 may be a potential therapeutic target for BC in the future.

## Data Availability

The data presented in this study are available on request from the corresponding author. Data may be available upon request to interested researchers.

## Supplementary Materials

The following supporting information can be downloaded at: https://www.mdpi.com/article/10.3390/ijms23042154/s1.
