# Supplementary material for: Fatty Acid Binding Protein 6 Inhibition Decreases Cell Cycle Progression, Migration and Autophagy in Bladder Cancers
Source: Int J Mol Sci. 2022 Feb 15;23(4):2154. doi: 10.3390/ijms23042154 (PMC8878685; doi:10.3390/ijms23042154)
Supplement: Supplementary file 1 [file ijms-23-02154-s001.zip › ijms-1543759-supplementary.pdf]

International Journal of
Molecular Sciences

MDPI

Supplementary Figures

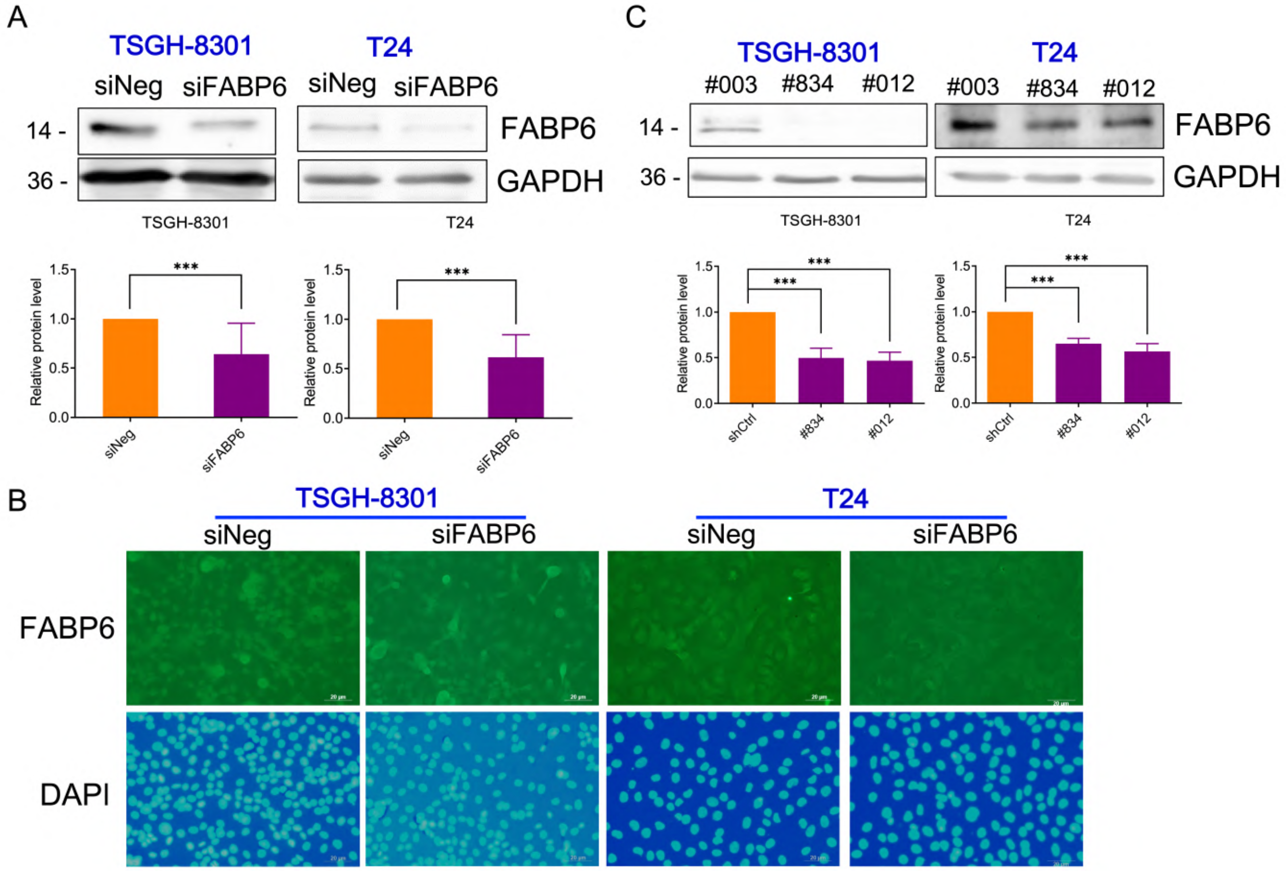

Supplementary Figure S1. Reduced FABP6 mRNA and protein expression in TSGH-8301 and T24 BC cells. (A) Quantitative analysis of FABP6 protein expression by western blotting in TSGH-8301 and T24 cells 72 h post-transfection. GAPDH was a loading control. (B) Immunostaining of FABP6 after 72 h post-transfection. (C) Knockdown effects of FABP6 were analyzed by western blotting in TSGH-8301 and T24 cells. Two cell lines were infected by scramble control lentivirus and another two shFABP6 sequences, #834 and #012, were selected. GAPDH was used as a loading control. ***, $p < 0.001$ compared to siNegtive group or #003 scramble control group.

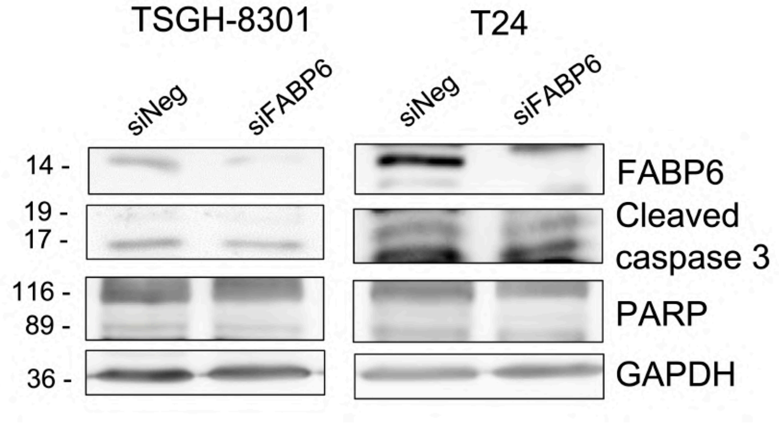

Supplementary Figure S2. Knockdown of FABP6 had no effect on apoptotic-related proteins. Western blotting analysis of caspase 3 and PARP expression 72 h after FABP6 knockdown in TSGH-8301 (left) and T24 (right) cells. GAPDH was used as a loading control.

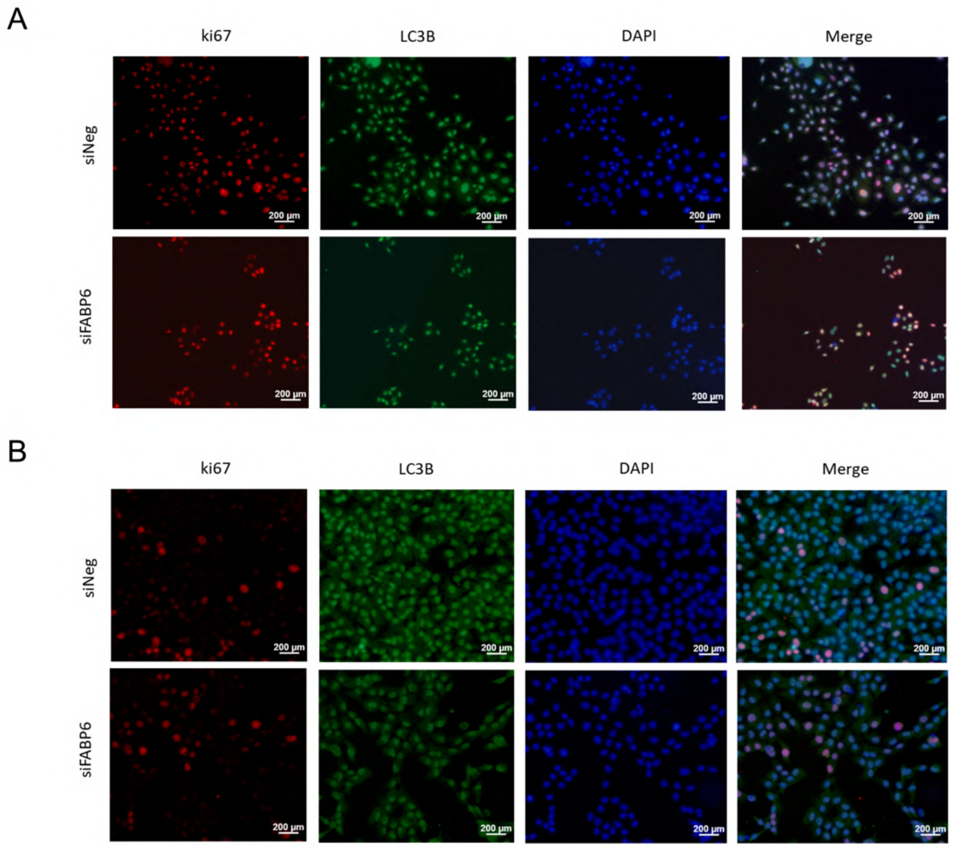

Supplementary Figure S3. Ki67 and LC3B immunostaining after FABP6 knockdown. Immunostaining of Ki67 (Red) and LC3B (Green) after 72 h post-transfection. Scale bar = 200 µm.

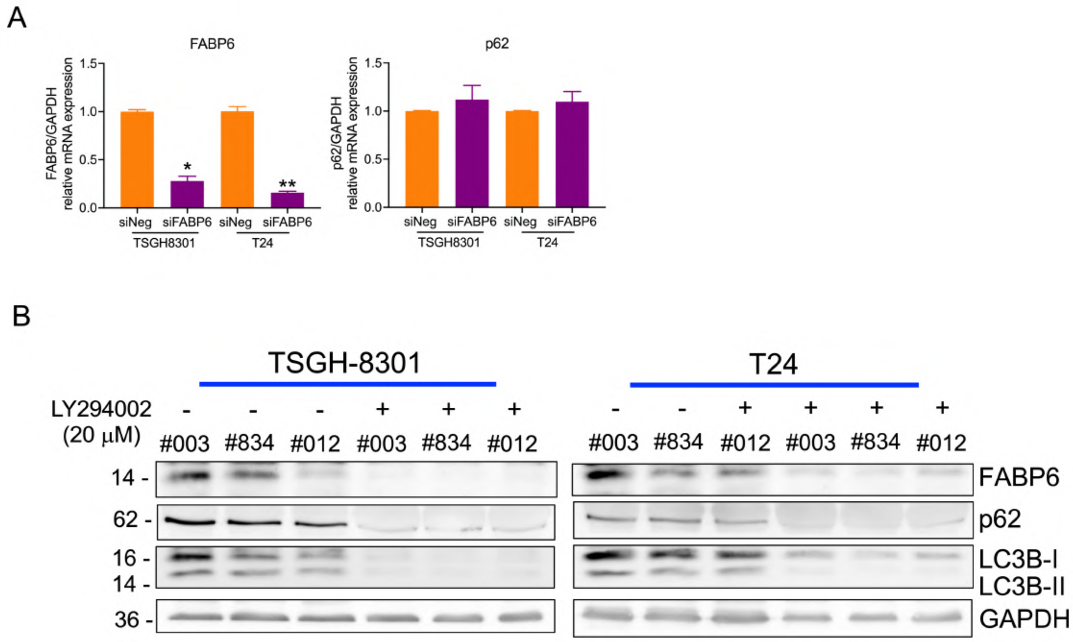

Supplementary Figure S4. The effect of LY294002 on p62 and LC3B after FABP6 inhibition. (A) Quantitative real-time PCR was performed after siFABP6 transfected for 72 h. The mRNA of FABP6 and p62 was evaluated in TSGH-8301 and T24 cells. (B) Quantitative analysis of p62 and LCB-II protein expression by western blotting in TSGH-8301 and T24 cells. GAPDH was a loading control. Two cell lines were infected by scramble control lentivirus (#003) and another two shFABP6 sequences, #834 and #012, were selected.

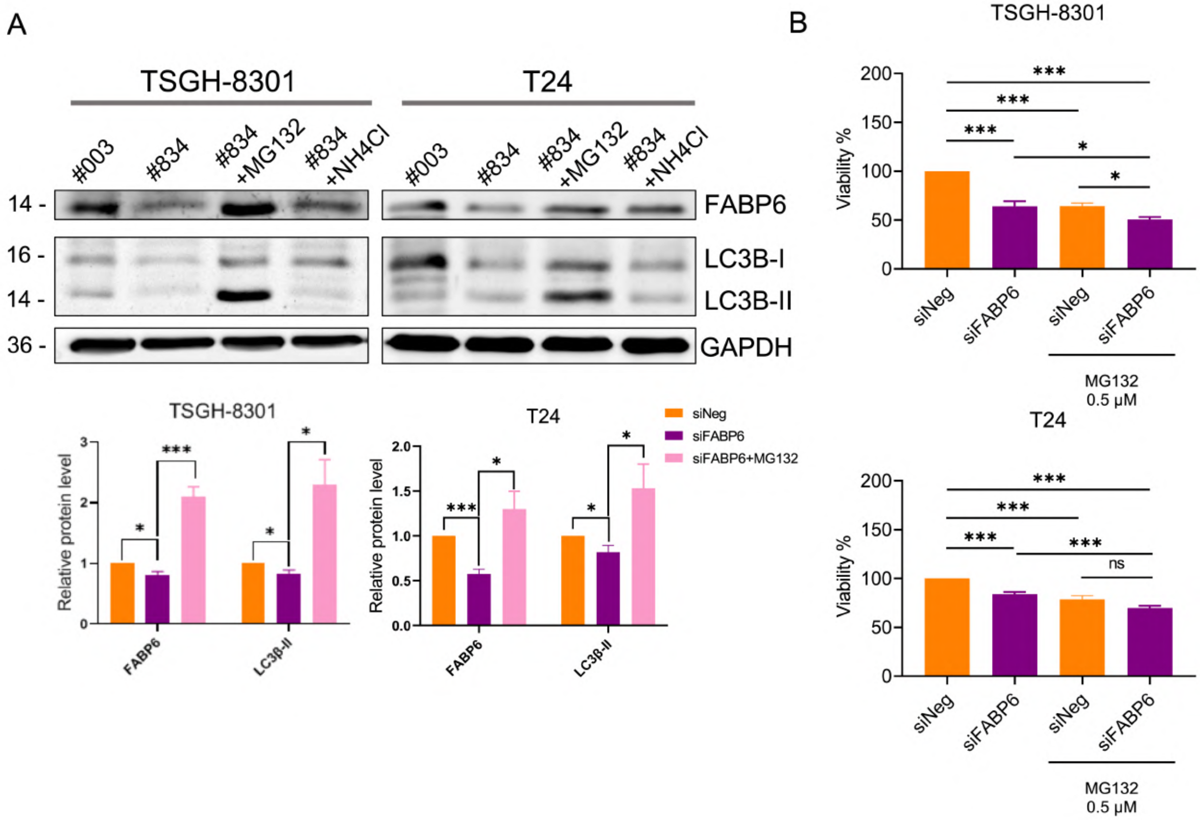

Supplementary Figure S5. Proteasome inhibitor MG132 treatment recovered autophagy markers after FABP6 knockdown. (A) In both cell lines showed reduced FABP6 and LC3B-II was recovered by MG132 treatment. (B)MTT assay analysis of proteasome inhibitor MG132 and lysosomal inhibitor NH4Cl treatment after knockdown of FABP6 for 72 h. These data are means ±"SEM " from at least three independent experiments. P value, one-way ANOVA with two tailed t-test. *, $p < 0.05$; **, $p < 0.01$; ***, $p < 0.001$.

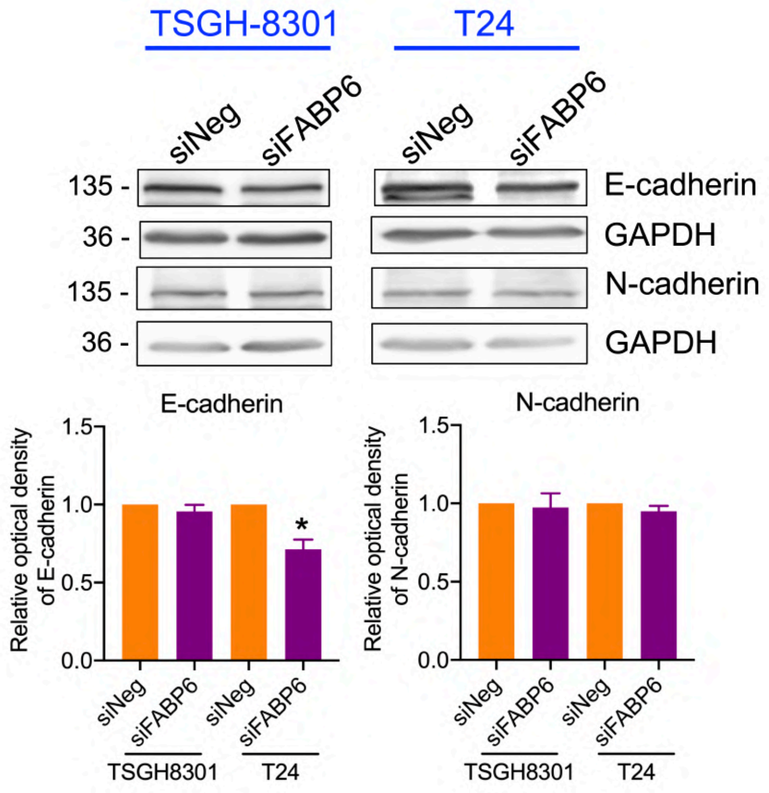

Supplementary Figure S6. The effect of E-cadherin and N-cadherin after FABP6 knockdown in BC cells. Quantitative analysis of E-cadherin and N-cadherin protein expression by western blotting in TSGH-8301 and T24 cells 72 h post-transfection. GAPDH was a loading control. *, $p < 0.05$ compared to siNegtive group.

Supplementary Table S1. The sequences of siRNAs targeting FABP6

Name	sequence	Target
D-00885-01	5’-GAAGAAUUAUGAUGAGUUC-3’	FABP6
D-00885-02	5’-GCGGGAAGCUGGUGGUGAA-3’	FABP6
D-00885-03	5’-GGGCAGGACUUCACUUGGU-3’	FABP6
D-00885-04	5’-GCAAGGAAAGCAACAUACA-3’	FABP6
D-001206-13	5’-UAGCGACUAAACACAUCAA-3’,	non-target
	5’-UAAGGCUAUGAAGAGAUAC-3’,	non-target
	5’-AUGUAUUGGCCUGUAUUAG-3’,	non-target
	5’-AUGAACGUGAAUGCUCAA-3’	non-target

Supplementary Table S2. The sequences of shRNAs targeting FABP6

Name	Sequences
**Scramble #003** (ASN0000000003)	**5’-CCGGCCTAAGGTTAAGTCGCCCTCGCTCGAGCGAGGGCGACTTAACCTTAGGTTTTT-3’**
**shFABP6 #834** (TRCN0000419834)	**5’-CCGGGAATTATGATGAGTTCATGAACTCGAGTTCATGAACCTCATCATAATTCTTTTT-3’**
**shFABP6 #012 (**TRCN0000447012)	**5’-CCGGTATGAGCGCGTGAGCAAGAGACTCGAGTCTCTTGCTCACGCGCTCATATTTTT-3’**

Supplementary Table S3. The information of antibody

Name	Species	Brand	Cat NO.
GAPDH	Rb	CST	5174S
Atg12	Rb	CST	2010P
Caspase3	Rb	CST	9661S
Caspase8	Ms	CST	9746S
CyclinA1+A2	Rb	Abcam	Ab185619
E-cadherin	Rb	BD	610181
FABP6	Rb	Novus	NBP1-32482
LC3B	Rb	CST	3868S
N-cadherin	MS	BD	610920
PARP	Rb	CST	9532S
p21	Rb	CST	2947T
p-p53	Rb	CST	9284S
p53	Rb	CST	9282S
p-p65 (S536)	Rb	Abcam	ab86299
p65	Rb	CST	8242S
p-ERK	Rb	CST	4377S
ERK1/2	Rb	CST	9102S
p-p38	Ms	Millipore	MABS64
p38	Rb	Abcam	ab7952
p-mTOR	Rb	CST	5536S
mTOR	Rb	CST	2972S
p-AKT (T308)	Rb	CST	13038S
AKT	Rb	Epitomics	1085-1
RXR α	Rb	CST	3085
PPAR γ	Rb	CST	2435
